# DNA- and RNA-SIP Reveal *Nitrospira* spp. as Key Drivers of Nitrification in Groundwater-Fed Biofilters

**DOI:** 10.1128/mBio.01870-19

**Published:** 2019-11-05

**Authors:** Arda Gülay, S. Jane Fowler, Karolina Tatari, Bo Thamdrup, Hans-Jørgen Albrechtsen, Waleed Abu Al-Soud, Søren J. Sørensen, Barth F. Smets

**Affiliations:** a Department of Environmental Engineering, Technical University of Denmark, Lyngby, Denmark; b Department of Biology, University of Copenhagen, Copenhagen, Denmark; c Nordic Center for Earth Evolution, Department of Biology, University of Southern Denmark, Odense, Denmark; d Department of Organismic and Evolutionary Biology, Harvard University, Cambridge, Massachusetts, USA; University of Oklahoma

**Keywords:** nitrification, comammox, *Nitrospira*, DNA SIP, RNA SIP

## Abstract

Nitrification, the oxidative process converting ammonia to nitrite and nitrate, is driven by microbes and plays a central role in the global nitrogen cycle. Our earlier investigations based on 16S rRNA and *amoA* amplicon analysis, *amoA* quantitative PCR and metagenomics of groundwater-fed biofilters indicated a consistently high abundance of comammox *Nitrospira*. Here, we hypothesized that these nonclassical nitrifiers drive ammonia-N oxidation. Hence, we used DNA and RNA stable isotope probing (SIP) coupled with 16S rRNA amplicon sequencing to identify the active members in the biofilter community when subjected to a continuous supply of NH_4_^+^ or NO_2_^−^ in the presence of ^13^C-HCO_3_^−^ (labeled) or ^12^C-HCO_3_^−^ (unlabeled). Allylthiourea (ATU) and sodium chlorate were added to inhibit autotrophic ammonia- and nitrite-oxidizing bacteria, respectively. Our results confirmed that lineage II *Nitrospira* dominated ammonia oxidation in the biofilter community. A total of 78 (8 by RNA-SIP and 70 by DNA-SIP) and 96 (25 by RNA-SIP and 71 by DNA-SIP) *Nitrospira* phylotypes (at 99% 16S rRNA sequence similarity) were identified as complete ammonia- and nitrite-oxidizing, respectively. We also detected significant HCO_3_^−^ uptake by *Acidobacteria* subgroup10, *Pedomicrobium*, *Rhizobacter*, and *Acidovorax* under conditions that favored ammonia oxidation. Canonical *Nitrospira* alone drove nitrite oxidation in the biofilter community, and activity of archaeal ammonia-oxidizing taxa was not detected in the SIP fractions. This study provides the first *in situ* evidence of ammonia oxidation by comammox *Nitrospira* in an ecologically relevant complex microbiome.

## Abstract (precis)

With this study we provide the first *in situ* evidence of ecologically relevant ammonia oxidation by comammox *Nitrospira* in a complex microbiome and document an unexpectedly high H_13_CO_3_^−^ uptake and growth of proteobacterial and acidobacterial taxa under ammonia selectivity. This finding raises the question of whether comammox *Nitrospira* is an equally important ammonia oxidizer in other environments.

## INTRODUCTION

Nitrification, the stepwise oxidation of ammonia (NH_3_) to nitrite (NO_2_^−^) and nitrate (NO_3_^−^), supplies the substrates for processes that initiate the loss of reactive nitrogen from the biosphere as N_2_. Understanding the organisms and environmental controls that drive nitrification is important as it controls global homeostasis of the N cycle. In engineered environments, complete nitrification is often desired: this is essential when waters are prepared and distributed for human consumption. Residual NH_3_ or NO_2_^−^—the result of incomplete nitrification—renders the water biologically unstable and unsafe for human consumption. Hence, biological systems for source water treatment are contingent on nitrifying prokaryotes. Based on evolutionarily conserved taxonomic (small subunit, 16S rRNA) and functional (e.g., ammonia monooxygenase [*amoA*]) gene surveys, *Nitrosomonas* (1–4), *Nitrosoarchaeum*, and *Nitrososphaera* have been identified as the abundant ammonia oxidizing prokaryotes (AOPs) and *Nitrospira* (5, 6) as the abundant nitrite-oxidizing prokaryotes (NOPs) in drinking water treatment systems, consistent with the classical assumption of division of labor in the two nitrification steps.

Our previous studies on rapid gravity sand filters (RGSFs), used in potable water preparation from groundwater, revealed nitrifying microbial communities in which *Nitrospira* is far more abundant than *Nitrosomonas* (7), with several *Nitrospira* genomes containing genes for ammonia oxidation (8), and with an abundance of comammox (complete ammonia-oxidizing) *amoA* over ammonia-oxidizing bacterial (AOB) *amoA* genes (9). Together with the concurrent discovery of comammox *Nitrospira* strains by others (10–12), this suggested that comammox *Nitrospira* may drive ammonia oxidation in the examined groundwater-fed RGSFs. In addition, like *Nitrospira*, several acidobacterial, and gamma- and alphaproteobacterial taxa were at consistently higher abundance than *Nitrosomonas*, raising questions about their potential role in nitrification, as NH_3_ is the primary growth substrate entering the filters (7, 13–15). Identifying the active ammonia- and nitrite-oxidizing organisms is essential not only for engineering purposes, but also for understanding the niches and biodiversity of nitrifiers. There has been a rapidly increasing documentation of global comammox *Nitrospira* occurrence across a myriad of habitats ranging from the subsurface, to soils, and sediments and from groundwaters to source and residual water treatment plants, but apparently excluding open oceanic waters (9, 16–19), with occasional abundances that exceed those of canonical AOB (9, 20, 21). Nonetheless, it is yet to be shown whether comammox *Nitrospira* truly drives ammonia oxidation in open oligotrophic freshwater and soil environments, their presumed preferred habitat based on genomic and physiological evidence (22, 23).

Here, we sought to identify the active ammonia and nitrite oxidizers in a groundwater-fed RGSF using RNA and DNA stable isotope probing (SIP) coupled to 16S rRNA amplicon sequencing. Lab-scale columns packed with filter material from a full-scale RGSF were fed with effluent water amended with NH_4_^+^ or NO_2_^−^ and with ^13^C-labeled or unlabeled HCO_3_^−^ for 15 days in the presence or absence of inhibitors of autotrophic ammonia and nitrite oxidation (24). Our findings indicate that *Nitrospira* drives both ammonia and nitrite oxidation. In addition, several other taxa take up substantial HCO_3_^−^ and their DNA and RNA increase in relative abundance when ammonia is the only provided energy source. This study provides the first *in situ* evidence of ammonia oxidation by comammox *Nitrospira* in an ecologically relevant complex microbiome.

## RESULTS

Four different experimental treatments were designed to identify the microbes involved in ammonia and nitrite oxidation (Table 1): (i) 71 μM NH_4_^+^ (columns 1 and 2), (ii) 71 μM NH_4_^+^ and 100 μM allylthiourea (ATU) (columns 3 and 4), (iii) 71 μM NO_2_^−^ (columns 5 and 6), and (iv) 71 μM NH_4_^+^ and 1 mM NaClO_3_ (columns 7 and 8). All columns were operated with an influent containing 100% ^13^C-labeled or 100% unlabeled bicarbonate for 15 days.

**TABLE 1:** Summary of experimental design, bulk ^13^C incorporation, substrate utilization and accumulation levels, and sequenced samples

Run and column^*a*^	N source	C source (^12^C or ^13^C)	Inhibitor	^13^C/^12^C ratio^*c*^	NH_4_^+^ removal (%)^*b*^	NO_2_^−^ removal (%)^*b*^	NO_3_^−^ accretion (%)^*b*^	Total DNA	Total RNA	SIP
Run 1										
Column 1	NH_4_^+^	H^13^CO_3_^−^		279	99 ± 1	100 ± 0	88 ± 32	**+**	**+**	**+**
Column 2	NH_4_^+^	HCO_3_^−^			98 ± 3	100 ± 0	82 ± 28	**+**	**+**	**+**
Column 3	NH_4_^+^	HCO_3_^−^	ATU		19 ± 15	101 ± 2	ND	**+**	**+**	**+**
Column 4	NH_4_^+^	H^13^CO_3_^−^	ATU	54	11 ± 15	99 ± 6	ND	**+**	**+**	**+**

Run 2										
Column 5	NO_2_^−^	H^13^CO_3_^−^		89	NA	88 ± 1	99 ± 34	**+**	**+**	**+**
Column 6	NO_2_^−^	HCO_3_^−^			NA	92 ± 3	62 ± 36	**+**	**+**	**+**
Column 7	NH_4_^+^	HCO_3_^−^	ClO_3_^−^		11 ± 5	70 ± 44	ND	**+**	**+**	**+**
Column 8	NH_4_^+^	H^13^CO_3_^−^	ClO_3_^−^	63	6 ± 7	97 ± 28	ND	**+**	**+**	**+**

a Run 1 was initiated with inoculum 1, and run 2 was initiated with inoculum 2.

b Removal and accumulation rates were estimated from daily NH_4_^+^, NO_2_^−^, and NO_3_^−^ measurements. NO_2_^−^ removal was calculated based on ammonium removed (except for columns 5 and 6, where it was based on influent nitrite). NO_3_^−^ accretion was calculated based on ammonium removed (except for columns 5 and 6, where it was based on nitrite removed). ND, differences between influent and effluent NO_3_^−^ concentrations were not significant, and accretion could not be calculated. NA, not applicable.

c Bulk ratio in columns after 15 days, as determined by EA-IRMS.

In columns 1 (H^13^CO_3_^−^) and 2 (HCO_3_^−^), the full-scale conditions were mimicked, with the aim to elucidate the complete *in situ* food web related to nitrification. In columns 3 (HCO_3_^−^) and 4 (H^13^CO_3_^−^), ATU was used to suppress bacterial ammonia oxidation while feeding at the same NH_4_^+^ loading as in columns 1 and 2 (25). Complete inhibition of bacterial ammonia oxidation with ATU has been observed at ATU concentrations of 8 to 86 μM (26), while archaeal ammonia oxidation is less sensitive to ATU (27). The mechanism of ATU inhibition in AOB is proposed to be chelation of the Cu^2+^ from the active site in the AMO enzyme (24). To identify taxa associated with nitrite oxidation, NO_2_^−^ was fed to columns 5 (H^13^CO_3_^−^) and 6 (HCO_3_^−^). In columns 8 (H^13^CO_3_^−^) and 7 (HCO_3_^−^), ClO_3_^−^ was used to inhibit nitrite oxidation under NH_4_^+^ feeding, with the aim to identify the taxa solely associated with NH_4_^+^ oxidation (25). Chlorate is commonly used as a selective inhibitor for nitrite oxidation, as it is reduced by reverse activity of the nitrite oxidoreductase to the toxic chlorite (ClO_2_^−^) (28, 29).

### Physiological activity.

In the 71 μM NH_4_^+^-fed treatments, complete NH_4_^+^ removal (99%) was observed without inhibitor addition, while NH_4_^+^ removal ranged from 11% to 19% with ATU amendment. (Table 1; see Fig. S3A in the supplemental material). Inhibitor addition also significantly reduced overall ^13^C incorporation. Columns fed with NH_4_^+^, NH_4_^+^-ATU, and NO_2_^−^ all had similarly high degrees of NO_2_^−^ removal ranging from 88% to 100%. In the 1 mM ClO_3_^−^-amended columns, NH_4_^+^ removal was severely inhibited (6% to 11%); removal of formed NO_2_^−^ continued (from 70% to 54%), although accumulation of NO_3_^−^ could not be detected. Nitrogen mass balances, based on influent and effluent NH_4_^+^, NO_2_^−^, and NO_3_^−^ concentrations closed for most experimental runs, minimizing the possibility of additional nitrogen cycling; N loss was only observed in the ATU-supplemented columns (columns 3 and 4) with ongoing treatment (Table 1; Fig. S3B and C).

10.1128/mBio.01870-19.4 FIG S3 (A) Changes in NH_4_^+^, NO_2_^−^, and NO_3_^−^ concentrations in all columns, (B and C) Difference in total N concentration in the influent and the effluent (σ = effluent N − influent N) calculated with equation 1 given in Text S1. (B) σ values as a function of operation days for columns 1, 2, 3, and 4 (NH_4_^+^ and NH_4_^+^-ATU treatments). (C) σ values as a function of operation days for columns 5, 6, 7, and 8 (NO_2_^−^ and NH_4_^+^-ClO_3_^−^ treatments). The significance of the difference between influent and effluent total N was evaluated using a two-tailed *t* test (significance level of 0.05). Differences were rejected for columns 1 and 2 and 5 to 8. In columns 3 and 4, the mass balance indicates N loss during the last sampling points. Download FIG S3, PDF file, 0.2 MB. Copyright © 2019 Gülay et al. 2019 Gülay et al. This content is distributed under the terms of the Creative Commons Attribution 4.0 International license.

10.1128/mBio.01870-19.1 TEXT S1 Supplemental materials and methods. Download Text S1, PDF file, 0.2 MB. Copyright © 2019 Gülay et al. 2019 Gülay et al. This content is distributed under the terms of the Creative Commons Attribution 4.0 International license.

### Detection of ^13^C-labeled taxa from DNA- and RNA-SIP.

DNA and RNA, extracted from column samples taken at the end of the experiments, were subjected to equilibrium density centrifugation, gradient fractionation, and 16S rRNA gene amplification. A total of 147 and 65 gradient fractions from DNA-SIP and RNA-SIP were sequenced using the Illumina Miseq and 454 pyrosequencing platforms, respectively (see Fig. S1a and b in the supplemental material). Operational taxonomic units (OTUs) were defined at 99% similarity, to minimize the effect of microdiversity, as similarities of 98.7% and lower represent the taxonomic levels of species, genus, and higher (30).

10.1128/mBio.01870-19.2 FIG S1 (A) Profile of DNA concentration across the CsCl buoyant density gradient obtained from DNA-SIP fractions after 15 days of operation. (B) Profile of RNA concentration across the CsTFA buoyant density gradient obtained from RNA-SIP fractions after 15 days of operation. (C) Simulated and actual RNA concentration profiles across fractions based on a normal distribution, following the procedure by Zemb et al. (1). Download FIG S1, PDF file, 0.6 MB. Copyright © 2019 Gülay et al. 2019 Gülay et al. This content is distributed under the terms of the Creative Commons Attribution 4.0 International license.

We first examined the incorporation of ^13^C in OTUs in all treatments by comparing replicate columns with H^12^CO_3_^−^ versus H^13^CO_3_^−^ amendment. In DNA-SIP, where all SIP fractions were sequenced, we calculated the average shift in buoyant density of each OTU based on its relative sequence abundance and buoyant density in all fractions (see equation 2 in Text S1 in the supplemental material). As only selected fractions were sequenced in RNA-SIP, the mean buoyant density of each OTU in treatments with H^12^CO_3_^−^ versus H^13^CO_3_^−^ amendment was calculated using the standard deviation of the RNA distribution across the buoyant density gradient (Fig. S1C), as described by Zemb et al. (31). The buoyant density shift of each OTU was then determined from the calculated mean buoyant density in the replicate columns of each treatment.

Among all detected OTUs (3,364,425), 4,075 and 5,045 in the NH_4_^+^ treatment, 4,133 and 5,155 in the NH_4_^+^ plus ATU treatment, 4,183 and 706 in the NO_2_^−^ treatment, and 44,916 and 52 in the NH_4_^+^-ClO_3_^−^-fed treatment showed a buoyant density shift (after filter 1; see Fig. S2 in the supplemental material) in the DNA- and RNA-SIP experiments, respectively. Only those OTUs that belonged to genera that contained OTUs that were ^13^C labeled by both RNA and DNA-SIP were retained (filter 2; Fig. S2). A bootstrap resampling of labeled OTUs within each genus was then used to estimate taxon-specific 90% confidence intervals (Cls) for the buoyant density shift of a labeled genus (filter 3; Text S1 and Fig. S2).

10.1128/mBio.01870-19.3 FIG S2 Sequential work flow to detect putative ammonia and nitrite oxidizers using RNA and DNA SIP. A detailed explanation of each filter step is given in Text S1. Download FIG S2, PDF file, 0.2 MB. Copyright © 2019 Gülay et al. 2019 Gülay et al. This content is distributed under the terms of the Creative Commons Attribution 4.0 International license.

Hence, after the 3rd filter step, 676 (57% DNA and 43% RNA of the total ^13^C-labeled OTUs, NH_4_^+^-fed treatment), 735 (67% DNA and 33% RNA, NH_4_^+^-ATU-fed treatment), 529 (65% DNA and 35% RNA, NO_2_^−^ treatment), and 43 (83% DNA and 16% RNA, NH_4_^+^-ClO_3_^−^-fed treatment) OTUs were retained as significantly labeled. The fractional ^13^C uptake of labeled OTUs was calculated by dividing the DNA and RNA buoyant density shift for each OTU by the total observed buoyant density shift for DNA and RNA, respectively. The abundances of labeled OTUs in the total community were estimated based on total (i.e., nonfractionated) DNA and rRNA extracts collected on day 15 (see Fig. S4A to D in the supplemental material).

10.1128/mBio.01870-19.5 FIG S4 16S rRNA-based phylogenetic tree of OTUs incorporating HCO_3_^−^ in DNA and RNA-SIP experiments of treatments consisting of (A) solely NH_4_^+^, (B) NH_4_^+^ plus ATU, (C) NH_4_^+^ plus ClO_3_^−^, or (D) solely NO_2_^−^. Peak heights on circles represent (i) relative abundance in total DNA (purple) and (ii) total RNA (red) after 15 days, as well as (iii) the ^13^C label percentage (orange). The scale bar represents 0.10 substitution per nucleotide position. Download FIG S4, PDF file, 1.1 MB. Copyright © 2019 Gülay et al. 2019 Gülay et al. This content is distributed under the terms of the Creative Commons Attribution 4.0 International license.

**FIG 1:**
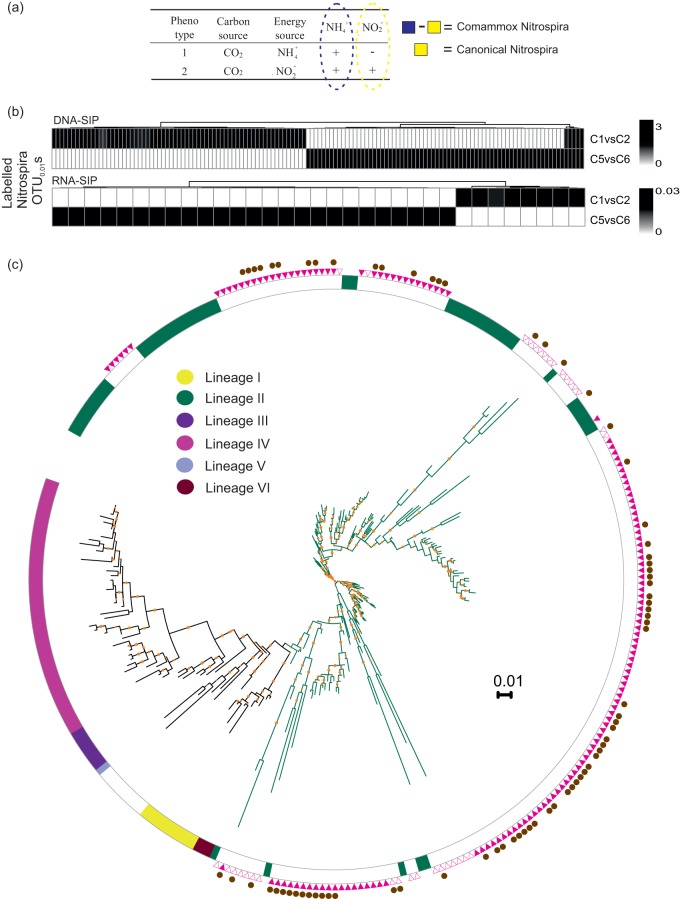
(a) Approach to identify ammonia- and nitrite-oxidizing *Nitrospira*. (b) Heat map of all identified labeled *Nitrospira* OTUs in columns fed with NH_4_^+^ and NO_2_^−^. (c) 16S rRNA-based phylogenetic tree of all identified labeled *Nitrospira* OTUs and published *Nitrospira* strains with known lineages. Open and filled triangles represent *Nitrospira* OTUs identified by RNA- and DNA-SIP, respectively. Comammox *Nitrospira* sequences are indicated by filled circles. Bootstrap values of >60% are shown by orange dots. The scale bar represents 0.01 substitution per nucleotide position.

In the NH_4_^+^-only fed treatment, ^13^C-labeled OTUs affiliated with 17 genera of the *Alpha*-, *Beta*-, and *Gammaproteobacteria*, *Nitrospira*, *Actinobacteria*, *Latescibacteria*, and *Acidobacteria* (Fig. S4A). Among them, the genus *Nitrospira* had the highest fraction of ^13^C uptake (32% and 1.1% for DNA-SIP and RNA-SIP, respectively) and highest relative abundance in the total DNA (26%). *Nitrosomonas* OTUs were also labeled but displayed low levels of ^13^C uptake (0.07% and 0.8% for DNA-SIP and RNA-SIP, respectively) and were at low abundance (0.15% and 0.18% in total DNA and RNA, respectively). Labeled rRNA, an approximation of metabolic activity, was distributed evenly between 5 different ^13^C-labeled genera, including *Woodsholea*, *Blastocatella*, subgroup 10 *Acidobacteria*, *Pedomicrobium*, and *Sphingomonas* (Fig. S4A). Although ammonia oxidation was severely inhibited in the NH_4_^+^-ATU-fed column (Fig. 1), OTUs in 15 genera incorporated ^13^C. These were identical to labeled OTUs in the NH_4_^+^-fed treatment, with the exception of OM27, *Rhizobacter*, *Variovorax* and uncultured representatives of the order *Xanthomonadales*, which were not labeled in the presence of ATU (Fig. S4B). *Azospira* incorporated H^13^CO_3_^−^ only in the presence of ATU. In the NH_4_^+^-ATU-fed treatment, *Nitrospira* (10% DNA-SIP, 4.7% RNA-SIP), *Pseudomonas* (3% DNA-SIP, 1% RNA-SIP), *Methyloglobulus* (2.7% DNA-SIP, 2.6% RNA-SIP), and *Blastocatella* (2.6% DNA-SIP, 3.1% RNA-SIP), incorporated the highest fraction of label, while *Sphingomonas* (1.8%) and *Woodsholea* (1.4%) were dominant in the total RNA pool (Fig. S4B). In the NH_4_^+^-ClO_3_^−^-amended columns, where both ammonia and nitrite oxidation were suppressed, OM27 (2.7% DNA, 2.6% RNA) and *Woodsholea* were the only taxa that assimilated significant amounts of HCO_3_^−^ (Fig. S4C).

In the NO_2_^−^-fed columns, 12 genera, belonging to Alphaproteobacteria (10% of H^13^CO_3_^−^ uptake in DNA-SIP, 23% of H^13^CO_3_^−^ uptake in RNA-SIP), Deltaproteobacteria (1% DNA-SIP, 21% RNA-SIP), and *Gammaproteobacteria* (6% DNA-SIP, 11% RNA-SIP), *Nitrospira* (71% DNA-SIP, 15% RNA-SIP), *Actinobacteria* (3% DNA-SIP, 7% RNA-SIP), *Latescibacteria* (0.5% DNA-SIP, 5% RNA-SIP), and *Acidobacteria* (6% DNA-SIP, 11% RNA-SIP), were labeled (after filter 3; Fig. S4D). *Nitrospira* had the highest number of labeled OTUs (a total of 96) and was responsible for the majority of the H^13^CO_3_^−^ uptake. After application of filters 4 through 6, only *Nitrospira* OTUs were retained and hence identified as the sole nitrite oxiders.

### Hypothesis: *Nitrospira* is an active ammonia oxidizer.

H^13^CO_3_^−^ was incorporated by *Nitrospira* in treatments fed with NH_4_^+^, NH_4_^+^-ATU, and NO_2_^−^ (after filter 3, Fig. S4A to D). The observed labeling in individual treatments does not indicate whether labeled *Nitrospira* OTUs are capable of ammonia oxidation because both ammonia and nitrite oxidation occur in NH_4_^+^ treatment. We therefore performed a binary comparison between labeled *Nitrospira* OTUs detected in the NH_4_^+^- versus NO_2_^−^-fed treatments (Fig. 1a). We assume that the labeled *Nitrospira* OTUs in NH_4_^+^-fed treatment would include both comammox and nitrite-oxidizing *Nitrospira*, while the NO_2_^−^-fed treatment would exclude comammox *Nitrospira* based on observation that comammox *Nitrospira* growth is not supported by oxidation of environmental nitrite in the absence of ammonia (11).

Heat maps of labeled *Nitrospira* OTUs (Fig. 1b) reveal that 8 (24%) and 70 (51%) OTUs are uniquely labeled in NH_4_^+^-fed treatment at the RNA and DNA levels, respectively, indicating that several ammonia-oxidizing *Nitrospira* strains are actively assimilating H^13^CO_3_^−^. A large number of labeled *Nitrospira* OTUs are unique to the NH_4_^+^-fed column, and few *Nitrospira* OTUs are shared between the NH_4_^+^- and NO_2_^−^-fed columns, suggesting that most comammox *Nitrospira* do not readily switch from ammonia oxidation to nitrite oxidation. A large number of OTUs were also uniquely labeled in the NO_2_^−^-amended columns (25 in RNA and 66 in DNA), which suggests that, in the NH_4_^+^-fed treatment, the produced NO_2_^−^ was not sufficient to achieve labeling of nitrite-oxidizing *Nitrospira* due to complete nitrification by commamox *Nitrospira*.

Based on their ^13^C labeling in NH_4_^+^- and NO_2_^−^-fed treatments, 78 (8 in RNA-SIP and 70 in DNA-SIP) and 96 (25 in RNA-SIP and 71 in DNA-SIP) *Nitrospira* OTUs were identified as complete ammonia and nitrite oxidizing, respectively (Fig. 1c). All labeled *Nitrospira* OTUs belonged to lineage II, which comprises both comammox and non-comammox types. No clear branching between comammox and nitrite-oxidizing phylotypes was observed from the tree topology.

### High H^13^CO_3_^−^ incorporation and growth by other bacteria.

**FIG 2:**
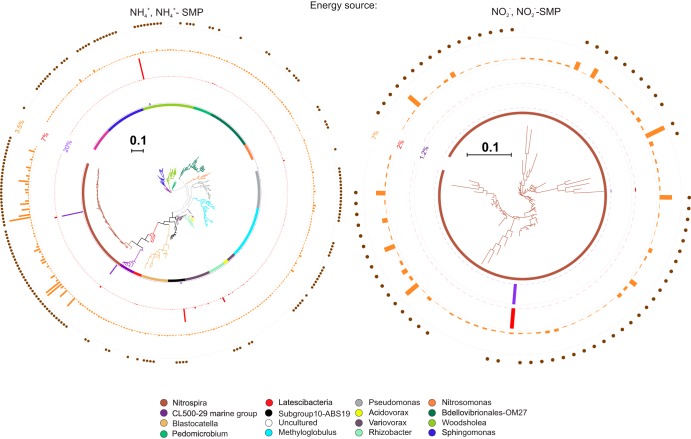
16S rRNA-based phylogenetic tree showing phylotypes incorporating H^13^CO_3_^−^ in DNA-SIP and RNA-SIP experiments selective for putative ammonia (left) and nitrite (right) oxidation. Peak heights on circles represent (i) relative abundance in total DNA (purple) and (ii) total RNA (red) after 15 days, as well as (iii) ^13^C label percentage (orange). The outer ring represents the OTUs retrieved from DNA-SIP (filled circles) or RNA-SIP (no circles). The scale bar represents 0.10 substitution per nucleotide position.

**FIG 3:**
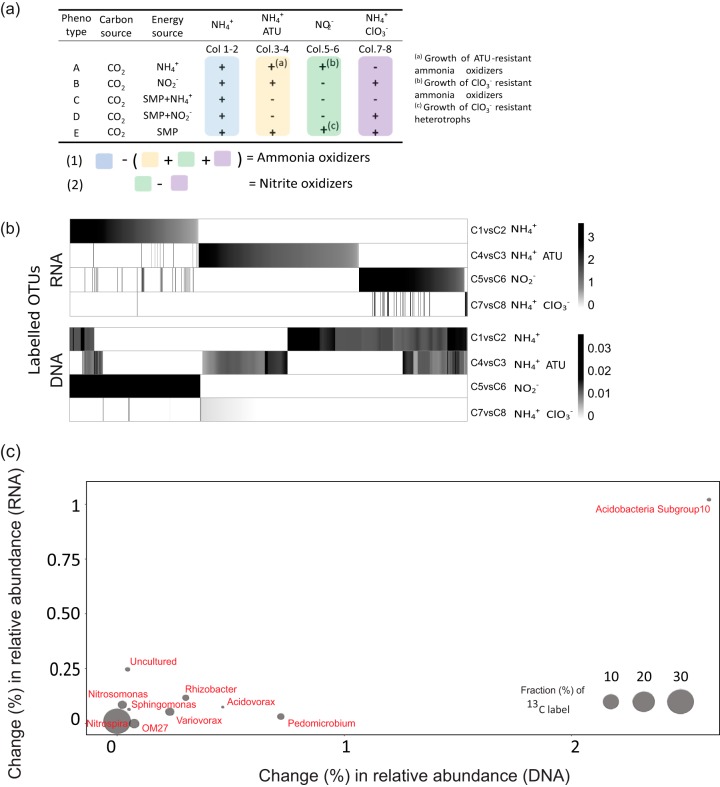
(a) Approach (filter 6; Text S1 and Fig. S3) to identify putative ammonia- and nitrite-oxidizing phylotypes. (b) Heat map of OTUs significantly labeled under NH_4_^+^, NO_2_^−^, NH_4_^+^-ATU, and NH_4_^+^-ClO_3_^−^ treatments. (c) Fold change in relative abundance in community DNA and RNA for taxa identified as putative ammonia oxidizers (as shown also in the left panel of Fig. 2).

In our previous 16S rRNA amplicon-based analysis of the same and related RGSF communities, members of the *Rhizobiales* (*Alphaproteobacteria*), and *Acidobacteria* were consistently more abundant than *Nitrosomonas*, where NH_4_^+^ is thought to be the largest source of energy available for microbial growth (7). We observed that some of these taxa incorporated ^13^HCO_3_^−^ in both RNA-SIP and DNA-SIP (after filter 6; Fig. 2, left). In addition, several OTUs displayed higher buoyant density shifts than *Nitrosomonas* in NH_4_^+^-fed columns (Fig. S4A). Finally, these OTUs also increased in relative abundance in both DNA and RNA over the course of the experiment (Fig. 3c).

Only *Nitrospira* was associated with both ammonia and nitrite oxidation (after filter 6; Fig. 2). Among the ^13^HCO_3_^−^-incorporating taxa in the presence of NH_4_^+^, subgroup 10 *Acidobacteria*, *Nitrospira* (*Nitrospira*), *Pedomicrobium* (*Alphaproteobacteria*), *Rhizobacter*, and *Acidovorax* (*Betaproteobacteria*) displayed higher shifts in relative RNA and DNA abundance compared to *Nitrosomonas* (Fig. 3c). With the exception of *Pseudomonas* (see Fig. S5A in the supplemental material), ^13^C-labeled genera in SIP columns (Fig. S4A to D) differed from the dominant genera in the feed water, itself the effluent from the full-scale biofilter. Hence, invasion from feed water communities did not cause the increased relative abundance of subgroup 10 *Acidobacteria*, *Pedomicrobium*, *Rhizobacter*, or *Acidovorax*.

10.1128/mBio.01870-19.6 FIG S5 (A) 16S rDNA-based relative abundances of dominant genera (%) in the communities in the feedwater to the test columns. (B) 16S rDNA (left)- and 16S rRNA (right)-based relative abundance of dominant genera (%) in communities extracted from the sand filter material at the onset (black lines) and after 15 days in columns 1 and 2 (green boxes) and columns 3 and 4 (red boxes). (C) 16S rDNA (left)- and 16S rRNA (right)-based relative abundance of dominant genera (%) in communities extracted from the sand filter material at the onset (black lines) and after 15 days in columns 5 and 6 (green boxes) and columns 7 and 8 (red boxes). Download FIG S5, PDF file, 0.4 MB. Copyright © 2019 Gülay et al. 2019 Gülay et al. This content is distributed under the terms of the Creative Commons Attribution 4.0 International license.

A metagenome, obtained from the same parent biofilter within the same year (2013 [8]) was further examined for its content of genes that are not canonical AOB *amoA*, canonical AOA *amoA*, *or* comammox *Nitrospira amoA*, nor methanotrophic *pmoA* (8) (see Fig. S6 in the supplemental material) but showed homology with genes encoding AMOA protein family fragments from putative heterotrophic nitrifiers (PF05145 and IPR017516 from the Pfam and InterPro databases, respectively). Twenty-nine unique *amoA* gene fragments matching the PF05145 model were aligned with reference putative heterotrophic *amoA* gene fragments (see Fig. S7 in the supplemental material). However, no *amoB* or *amoC* genes were found on any of the contigs carrying these atypical putative *amoA* gene fragments (Fig. S7); furthermore, no similar gene synteny was detected between the other genes of PF05145 *amoA*-containing contigs and our metagenome *amoA*-containing contigs (Fig. S7).

10.1128/mBio.01870-19.7 FIG S6 Phylogeny of putative heterotrophic *amoA* sequences retrieved from the metagenome and reference sequences obtained from (PF05145); the taxonomy of the metagenome-derived *amoA* sequences was inferred from the lowest common ancestor (LCA) of all of the genes on the respective contig. Download FIG S6, PDF file, 0.1 MB. Copyright © 2019 Gülay et al. 2019 Gülay et al. This content is distributed under the terms of the Creative Commons Attribution 4.0 International license.

10.1128/mBio.01870-19.8 FIG S7 Gene synteny on some of the contigs on which the putative heterotrophic *amoA* sequences (PF051459) were retrieved as well as gene synteny near reference sequences of *Pseudomonas* strains. Download FIG S7, PDF file, 0.1 MB. Copyright © 2019 Gülay et al. 2019 Gülay et al. This content is distributed under the terms of the Creative Commons Attribution 4.0 International license.

The genus *Nitrospira* was the only taxon associated with nitrite oxidation (Fig. 2, right).

## DISCUSSION

Stable isotope probing has previously been used to identify active nitrifiers in sediments (32–34) and soils (35–38). In most studies, either DNA-SIP (35) or RNA-SIP (39) is applied individually, yet both are important to identify key catalysts (38). While DNA-SIP detects isotope incorporation into dividing cells, RNA-SIP detects active potentially slow- or nongrowing cells (40). By coupling SIP with next-generation sequencing (NGS), we improved taxonomic resolution and differentiated phylotypes in taxa with high microdiversity.

Here, we examined the assimilation of H^13^CO_3_^−^ coupled to nitrification in a RGSF using both RNA- and DNA-SIP. OTUs incorporating ^13^C isotope label in the different treatments were unambiguously identified as those displaying significant buoyant density shifts between the H^12^CO_3_^−^ and the H^13^CO_3_^−^ replicates and were detected at high phylogenetic resolution (>99% pairwise identity [30]). A total of 200 gradient fractions were processed with sample size equalization.

Our results provide the first *in situ* physiological evidence of ecologically relevant NH_4_^+^ oxidation by comammox *Nitrospira* in any environment. Other reports of *in situ* activity are inferred from bulk observations (ammonium removal when comammox *Nitrospira* bacteria are more abundant than AOB or AOA [20, 41]) or from comammox-specific *amoA* transcript analysis (42). Our former observations that *Nitrospira* was more abundant than *Nitrosomonas* and the discovery that *Nitrospira* harbors the complete nitrification pathway in a full-scale RGSF microbiome (8, 10, 11) are thus directly linked to the ammonia-oxidizing activity of *Nitrospira* in this environment. *Nitrospira* was the only genus incorporating H^13^CO_3_^−^ in both NH_4_^+^-fed and NO_2_^−^-fed treatments, indicating that *Nitrospira* is the only genus oxidizing both environmental NH_4_^+^ and NO_2_^−^ in this system.

Ammonia- and nitrite-oxidizing phylotypes of *Nitrospira* were compared phylogenetically; the resulting 16S rRNA tree topology shows no clear evolutionary separation of comammox and canonical *Nitrospira*. This is in line with previous studies that show that comammox *Nitrospira* bacteria are not evolutionarily distant from known canonical *Nitrospira* (>99% 16S rRNA nucleotide identity) (10). Furthermore, our phylogenetic analysis shows that the labeled—both ammonia-oxidizing and nitrite-oxidizing—*Nitrospira* OTUs branch within *Nitrospira* sublineage II, as reported in previous studies (8, 10–12). We did not identify the *amoA* clade affiliation of the active comammox *Nitrospira* phylotypes in this study, although separate investigations on this and related RGSFs have indicated a dominance of *amoA* clade B over clade A comammox *Nitrospira* (9, 23).

It remains unclear whether comammox *Nitrospira* can switch between modes of ammonia and nitrite oxidation. However, the large numbers of *Nitrospira* phylotypes that were exclusively labeled in the NH_4_^+^- versus NO_2_^−^-fed columns, respectively, suggests that comammox *Nitrospira* may not prefer to oxidize external NO_2_^−^ alone, in agreement with observations in “*Candidatus* Nitrospira inopinata” (10, 11).

Although ClO_3_^−^ is a well-known competitive inhibitor of nitrite oxidoreductase (43), strong inhibition of both ammonia and nitrite oxidation was observed in NH_4_^+^-ClO_3_^−^-fed columns. We expect that the inhibition of nitrite oxidation in comammox *Nitrospira* would be caused by ClO_3_^−^ reduction to the toxic ClO_2_^−^, which would negatively affect overall metabolism in these organisms, including ammonia oxidation. Hence, it appears that the inhibitory effect of ClO_3_^−^ on ammonia oxidation provides preliminary support for the contribution of comammox *Nitrospira* to ammonia oxidation, as we observed before (25). In addition, ClO_2_^−^ may contribute to inhibition of other ammonia oxidizers as observed before (44). PTIO, an NO-chelating compound, has also been documented as a potent inhibitor of ammonia oxidation in “*Candidatus* Nitrospira inopinata,” although its selectivity is unclear (45). ATU significantly suppressed ammonia oxidation in NH_4_^+^-ATU-fed treatments, although the taxa assimilating ^13^HCO_3_^−^ did not change significantly compared to the NH_4_^+^-fed treatment, excluding *Azospira*. This similarity in labeled taxa in the ATU-fed treatment may indicate that the ammonia monooxygenase (AMO) of the major ammonia oxidizers in this environment may be less sensitive to ATU than AOB at the given concentrations, as previously observed for AOA (46).

No archaeal taxa were ^13^C labeled in any of the columns, although archaeal ammonia oxidizers (AOAs) are present, albeit at much lower abundance than *Nitrospira* (100- to 1,000-fold), in the RGSF used in this study (8, 13, 47). Columns were fed 71 μM NH_4_^+^ to mimic full-scale conditions (14), while bottom layers of the full-scale biofilter receive very low ammonium concentrations due to removal in the top layers (14, 15). The absence of AOAs in the ^13^C-labeled taxa may be due to their low initial abundances or the elevated NH_4_^+^ concentrations applied during the experiment, as AOAs may thrive better under conditions of reduced energy supply consistent with their elevated abundance at bottom layers of the examined RGSF (48–51), even though a recently isolated comammox strain of “*Candidatus* Nitrospira inopinata” displayed higher NH_4_^+^ affinity than many of the characterized AOAs (22).

After strong filters were applied to remove heterotrophic OTUs, we retain the following taxa with substantial H^13^CO_3_^−^ incorporation in the NH_4_^+^-fed treatments: *Acidobacteria* subgroup10, *Pedomicrobium*, *Rhizobacter*, and *Acidovorax*; these taxa also show a greater shift in relative abundance in DNA and RNA during the experiment than *Nitrosomonas* and *Nitrospira* OTUs (Fig. 3c; see Fig. S8 in the supplemental material). Most heterotrophic microbes can engage in CO_2_ assimilation via carboxylation reactions (52, 53). However, CO_2_ assimilation via anaplerotic metabolism (54) typically results in only 3 to 8% of the cellular carbon assimilated by heterotrophs, which would be insufficient for label detection by DNA-SIP (52, 55). Thus, heterotrophic carbon assimilation would not explain the greater extents of H^13^CO_3_^−^ incorporation (higher density shifts; see Fig. 2 and see Table S1, parts A and B, in the supplemental material) relative to *Nitrosomonas* OTUs, known ammonia oxidizers. Furthermore, the cellular mass and activity supported by cross-feeding decay products from autotrophs (56) would be significantly less than the chemolithoautotrophic biomass and activity itself. Thus, the observations of ^13^C-labeled genera with higher buoyant density shifts and higher shifts in DNA and RNA abundance shifts (Fig. 2 and 3) compared to *Nitrosomonas* and *Nitrospira* are difficult to explain by cross-feeding alone.

10.1128/mBio.01870-19.9 FIG S8 (A) Relative abundance shifts in 16S rDNA (left) and 16S rRNA (right) (%) of the most dominant genera between day 0 and day 15 in columns 1 and 2. (B) Relative abundance shifts in 16S rDNA (left) and 16S rRNA (right) (%) of the most dominant genera between day 0 and day 15 in columns 3 and 4. (C) Relative abundance shifts in 16S rDNA (left) and 16S rRNA (right) (%) of the most dominant genera between day 0 and day 15 in columns 5 and 6. (D) Relative abundance shifts in 16S rDNA (left) and 16S rRNA (right) (%) of the most dominant genera between day 0 and day 15 in columns 7 and 8. Download FIG S8, PDF file, 0.6 MB. Copyright © 2019 Gülay et al. 2019 Gülay et al. This content is distributed under the terms of the Creative Commons Attribution 4.0 International license.

Can a plausible explanation for the high H^13^CO_3_^−^ assimilation of these taxa be nitrification? An earlier metagenome from the same parent material revealed the presence of putative *amoA* genes (Table S1, part C) that could not be classified as *amoA* from canonical AOB, canonical AOAs, or comammox *Nitrospira* (8) (Fig. S6), yet were phylogenetically related to PF05145, purported to contain AMOA-encoding genes in heterotrophic bacteria (57). In addition, the phylogeny of 10/30 of these aberrant *amoA* genes indicated their presence, among others, in *Hyphomicrobiaceae* and *Comamonadaceae*. Of the highly labeled taxa (in both RNA-SIP, and DNA-SIP) in the NH_4_^+^-fed treatment, *Pedomicrobium*, and *Acidovorax* (but not *Acidobacteria* subgroup 10 or *Rhizobacter*) belong to the *Hyphomicrobioaceae* and *Comamondaceae*. While it is tempting to speculate that we have identified novel ammonia-oxidizing bacteria, we were unable to identify additional *amo* genes that would constitute a complete *amo* operon on any of the metagenomic contigs. In addition, recent doubt has been cast on the assignment of PF05145 as encoding a putative ammonia monooxygenase (58), and careful physiological or genomic evidence of heterotrophic nitrification remains elusive (59). On the other hand, some of the acidobacterial metagenome-assembled genomes (MAGs) that were retrieved from the studied RGSF metagenome contained CO_2_ fixation pathways (i.e., CG15 encoded a near-complete reductive tricarboxylic acid [rTCA] pathway [8]).

The second step of nitrification, the oxidation of nitrite to nitrate, is known to be performed by nitrite-oxidizing chemolithoautotrophs such as *Nitrotoga*, *Nitrospina*, *Nitrobacter*, *Nitrolancea*, and *Nitrospira* (60–62), which use nitrite oxidoreductase (NXR) as the key enzyme. The known autotrophic nitrite oxidizer *Nitrospira* was identified as the only active nitrite oxidizer in the studied system.

In summary, comammox *Nitrospira* and *Nitrosomonas* are the chemolithoautotrophic drivers of ammonia oxidation in the groundwater-fed biofilter, and comammox *Nitrospira* make the greatest contribution. The fundamental niche of comammox *Nitrospira*, however, remains poorly defined. While kinetics and modeling suggest that these bacteria thrive in environments with low ammonium concentrations (22, 63), as observed in this study, they are equally abundant in some environments with higher ammonium content, such as fertilized soils and wastewater treatment systems (64). AOAs did not contribute significantly to nitrification, and *Nitrospira* bacteria were the only nitrite oxidizers identified in this environment. Hence, we provide the first *in situ* evidence of ecologically relevant ammonia oxidation by comammox *Nitrospira* in a complex microbiome and document an unexpectedly high H^13^CO_3_^−^ uptake and growth of proteobacterial and acidobacterial taxa under ammonium selectivity.

## MATERIALS AND METHODS

### Sampling sites and procedure.

Filter material samples were collected from a rapid gravity sand filter (biofilter) at the Islevbro waterworks (Rødovre, Denmark) in May 2013. The influent and effluent water quality is reported elsewhere (13, 14, 65). Filter material was collected from three random horizontal locations of the biofilter using a hand-pushed core sampler. From the extracted filter material core, the top 10 cm was aseptically segregated on site and stored on ice for further use. A portion was frozen on-site in liquid nitrogen for RNA extraction.

### Column experiments and stable isotope labeling.

Experiments were conducted using a continuous-flow lab-scale system consisting of glass columns (2.6 cm diameter, 6 cm long) filled with parent filter material (26.5 cm^3^) as described previously (14). Effluent water from the investigated waterworks was used as the medium in all experiments to avoid interference of other autotrophic processes and approximate full-scale conditions.

The experimental design consisted of 4 treatments applied to columns fed with ^13^C-labeled or unlabeled HCO_3_^−^ (at 1 mM). The influent and effluent pHs of all treatments were 7.5 to 7.6. The experiments were organized in two phases of 4 columns each; filter material was sampled for DNA and RNA extraction just before the onset of each experimental phase. In the 4 treatments, the influent waters were spiked with (i) NH_4_^+^ (NH_4_Cl at 1 mg/liter N [71 μM]; Sigma-Aldrich, 254134), (ii) NH_4_^+^ and ATU (*N*-allylthiourea at 100 μM; Merck Chemicals, 808158), (iii) NO_2_^−^ (NaNO_2_ at 1 mg/liter N [71 μM]; Sigma-Aldrich, S2252), or (iv) NH_4_^+^ and ClO_3_^−^ (KClO_3_ at 1 mM; 99%, Sigma-Aldrich, 12634) (Table 1) (25). The applied flow rates (40 ml/h) and influent (NH_4_^+^ or NO_2_^−^) concentrations were set to match the volumetric NH_4_^+^-N loading rates (approximately 1.5 g N/m^3^/h) experienced by the full-scale parent biofilter (14). Test and control columns were operated for 15 days with continuous feeding to allow sufficient ^13^C label incorporation. Further details are given in Text S1.

### Analytical methods.

Column effluents were sampled daily, filtered (0.2-μm-pore cutoff), frozen and analyzed colorimetrically for NH_4_^+^ and NO_2_^−^ as described in Tatari et al. (25). Colorimetric analysis of ammonium in samples containing ATU underestimated the NH_4_^+^ concentration (25), and thus NH_4_^+^ in these samples was quantified by flow injection analysis (66). NO_3_^−^ was quantified by ion chromatography (Dionex, ICS 1500) with a device fitted with a guard column (Dionex, AG 22) and an analytical column (Dionex, Ion Pac AS22). NH_4_^+^ removal (%) was calculated by subtracting effluent from influent NH_4_^+^ concentration and normalizing for the influent NH_4_^+^ concentration. NO_2_^−^ removal (%) was calculated as the difference between produced NO_2_^−^ concentration and effluent NO_2_^−^ concentration, after correcting for trace NO_2_^−^ present in the water (ca. 0.3 μM NO_2_^−^) and normalization for the produced NO_2_^−^ concentration. The NO_2_^−^ produced by ammonia oxidation was estimated as the difference between influent and effluent NH_4_^+^ concentrations. NO_3_^−^ accumulation (%) was calculated from the difference between the effluent and influent NO_3_^−^ concentrations, normalized for the produced NO_3_^−^ concentration. The NO_3_^−^ produced was estimated as the difference between influent and effluent NH_4_^+^ (or NO_2_^−^ in the case of columns 5 and 6) concentrations.

### Nucleic acid extraction and SIP.

Filter material samples collected from the full-scale biofilter and the sacrificed columns were subject to DNA and RNA extraction. Genomic DNA was extracted from 0.5 g of drained filter material using the MP FastDNA Spin kit (MP Biomedicals, LLC, Solon, OH) according to manufacturer’s instructions. The concentration and purity of extracted DNA were checked by spectrophotometry (NanoDrop Technologies, Wilmington, DE). RNA was extracted from frozen filter material samples (−80°C) with a MoBio PowerSoil total RNA isolation kit (no. 12866-25) according to the manufacturer’s instructions. The RNA was further purified with a Qiagen AllPrep DNA/RNA minikit (Hilden, Germany) and quantified with a Ribogreen RNA-quantification kit (Invitrogen, Eugene, OR). Extracted rRNA (approximately 650 ng) was mixed well with cesium trifluoroacetate solution to achieve an initial density of 1.790 g/ml before ultracentrifugation at 38,400 rpm for 72 h at 20°C in a Beckman VTi 65.2 rotor (67). Centrifuged rRNA gradients were fractionated into 250-μl fractions, the buoyant density of each fraction was measured by refractometry, and rRNA was precipitated from fractions as described previously by Whiteley et al. (68). The concentration of purified RNA was determined using a Ribogreen RNA quantification kit.

Density gradient ultracentrifugation of DNA isolated from columns and full scale was performed according to Neufeld et al. (69). Briefly, 1.6 μg of DNA in CsCl with a final density of approximately 1.725 g/ml was subject to ultracentrifugation at 44,800 rpm for 44 h, 20°C in a Beckman ultracentrifuge with a Beckman VTi 65.2 rotor (Beckmann). Gradients were fractionated into 250-μl fractions, density was determined by refractometry, and DNA was recovered by precipitation with PEG. DNA concentration was determined using a Picogreen high-sensitivity double-stranded DNA (dsDNA) quantification kit (Invitrogen).

### PCR amplification and tag sequencing.

RNA samples purified from density gradient fractions and from sacrificed column experiments and full-scale biofilters were reverse transcribed using reverse primer 1492R with the Sensiscript reverse transcription (RT) kit (Qiagen) according to the manufacturer’s protocol. Ten nanograms of cDNA or DNA from direct DNA extracts (Table 1) was used to amplify the V3 and V4 regions of bacterial 16S rRNA genes using the Phusion (*Pfu*) DNA polymerase (Finnzymes, Finland) and 16S rRNA gene-targeted (rDNA) modified universal primers PRK341F and PRK806R (70). PCR was performed as described in reference 7. All fractions (a total of 145 [Fig. S1A]) from DNA-SIP and selected fractions (a total of 62 [Fig. S1B]) from RNA-SIP experiments were sequenced on an Illumina MiSeq and GS FLX pyrosequencing platform, respectively. Pyrosequencing was applied in a two-region 454 run on a 70-75 GS PicoTiterPlate using a Titanium kit (7); paired-end 16S rRNA amplicon sequencing was done on the Illumina MiSeq platform with MiSeq reagent kit v3 (2 × 301 bp; Illumina). All sequencing was performed at the National High-Throughput DNA Sequencing Center (Copenhagen, Denmark).

### Bioinformatic and statistical analysis.

All bioinformatic and statistical analyses are described in detail in Text S1. Briefly, raw 454 sequence data from RNA-SIP samples were quality-checked (denoised) with Ampliconnoise (71) and chimeras were removed with UCHIME (72) using default settings. Raw Miseq Illumina sequence data from DNA-SIP samples were quality-controlled with MOTHUR (73), and chimeras were removed with UCHIME (72) using a reference data set. Sequence libraries were combined and trimmed to 418 bp. All further sequence analyses were performed in QIIME 1.9.1 (74).

A total of six filter steps were applied to identify ammonia- and nitrite-oxidizing phylotypes (Fig. S2). Detailed steps are described in Text S1. OTUs incorporating H^13^CO_3_^−^ were determined by the following: (i) filter 1, comparing the mean buoyant density of each OTU in columns with and without ^13^C amendment (31); (ii) filter 2, identifying OTUs affiliated with genera that are present in both DNA and RNA SIP; and (iii) filter 3, selecting OTUs with buoyant density shifts higher than genus-specific 90% CIs for buoyant density shifts. The remaining filter steps were applied to assess ammonia and nitrite oxidizing phylotypes. Cross-feeders and taxa performing heterotrophic CO_2_ assimilation (i.e., carboxylation) were largely removed by (iv) filter 4, excluding OTUs with lower buoyant density shift than the maximum buoyant density shift value of labeled *Nitrosomonas* and *Nitrospira* OTUs, respectively, (v) filter 5, selecting the genera that contained OTUs in both RNA and DNA-SIP, and (vi) filter 6, comparing the labeled OTUs between treatments (NH_4_^+^ fed, NH_4_^+^-ATU fed, NO_2_^−^ fed, or NH_4_^+^-ClO_3_^−^ fed). To identify ammonia-oxidizing phylotypes, labeled OTUs in all treatments, excluding the one fed only with NH_4_^+^, were removed from the labeled OTU library of NH_4_^+^-fed treatment. To identify nitrite-oxidizing phylotypes, labeled OTUs in the treatment fed with ClO_3_^−^ were removed from the labeled OTU library of the only-NO_2_^−^-fed treatment.

As an additional step, detected genera were ranked according to the increase in their relative abundance in both total DNA and RNA from the beginning (day 0) to the end (day 15) of the experimental runs.

### Data availability.

R codes for all bioinformatics and statistics, including the detection of labeled OTUs in DNA- and RNA-SIP, can be found in https://github.com/ardagulay.

All sequence data have been deposited at NCBI GenBank under Biosample accession numbers from SAMN12227610 to SAMN12227705.

10.1128/mBio.01870-19.10 TABLE S1 (A) Genus-specific 90% CIs for the change in DNA buoyant density. (B) Genus-specific 90% CIs for the change in RNA buoyant density. (C) BLAST hits to the putative *amoA* sequences. Download Table S1, PDF file, 0.2 MB. Copyright © 2019 Gülay et al. 2019 Gülay et al. This content is distributed under the terms of the Creative Commons Attribution 4.0 International license.
