# Supplementary material for: DNA- and RNA-SIP Reveal Nitrospira spp. as Key Drivers of Nitrification in Groundwater-Fed Biofilters
Source: mBio. 2019 Nov 5;10(6):e01870-19. doi: 10.1128/mBio.01870-19 (PMC6831773; doi:10.1128/mBio.01870-19)
Supplement: FIG S2 [file mBio.01870-19-sf002.pdf]

DNA and RNA-SIP reveal Nitrospira spp. as key drivers of nitrification in groundwater-fed biofilters

Arda Gülay[1,4 *], Jane Fowler[1], Karolina Tatari[1], Bo Thamdrup[3], Hans-Jørgen Albrechtsen[1], Waleed Abu Al-Soud[2,] Søren J. Sørensen[2] and Barth F. Smets[1*]

[1] Department of Environmental Engineering, Technical University of Denmark, Building 113, Miljøvej, 2800 Kgs Lyngby, Denmark. **Phone:** +45 45251600. **FAX:** +45 45932850. **e-mail:** argl@env.dtu.dk, jfow@ env.dtu.dk, hana@env.dtu.dk, bfsm@env.dtu.dk*

[2] Department of Biology, University of Copenhagen, Universitetsparken 15, Building 1, 2100 Copenhagen, Denmark. **Phone:** +45 35323710. **FAX:** +45 35322128. **e-mail:** w.abualsoud@bio.ku.dk, sjs@bio.ku.dk

[3] Nordic Center for Earth Evolution, Department of Biology, University of Southern Denmark, Campusvej 55, 5230 Odense, Denmark. **Phone:** +45 35323710. **FAX:** +45 35322128. **e-mail:** bot@biology.sdu.dk

[4] Department of Organismic and Evolutionary Biology, Harvard University, Cambridge, MA, United States, 26 Oxford St, Cambridge, MA 02138, **Phone:** +1 (617)4951564. **e-mail:** ardagulay@fas.harvard.edu

Supplementary Figure 2

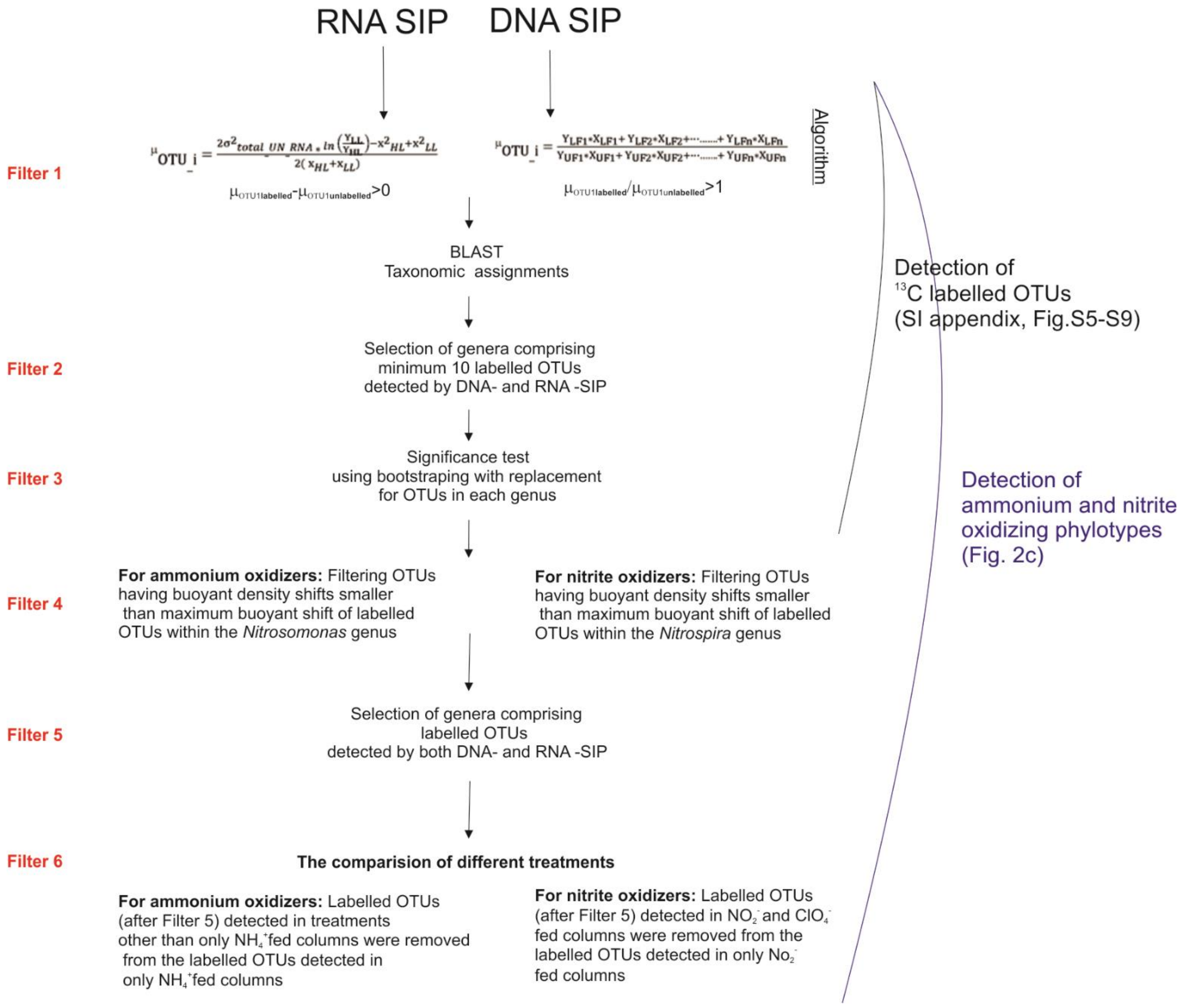

Fig.S2 Sequential work-flow to detect putative ammonium and nitrite oxidizers using RNA and DNA SIP. Detailed explanation of each filter step is given in Supplementary Materials and Methods.
