# Supplementary material for: DNA- and RNA-SIP Reveal Nitrospira spp. as Key Drivers of Nitrification in Groundwater-Fed Biofilters
Source: mBio. 2019 Nov 5;10(6):e01870-19. doi: 10.1128/mBio.01870-19 (PMC6831773; doi:10.1128/mBio.01870-19)
Supplement: TABLE S1 [file mBio.01870-19-st001.pdf]

DNA and RNA-SIP reveal Nitrospira spp. as key drivers of nitrification in groundwater-fed biofilters

Arda Gülay[1,4,*], Jane Fowler[1], Karolina Tatari, Bo Thamdrup[3], Hans-Jørgen Albrechtsen[1], Waleed Abu Al-Soud[2,] Søren J. Sørensen[2] and Barth F. Smets[1*]

Supplementary Table 1

Table S1-A Genus specific 90% CIs for the change in DNA buoyant density

Comparison	Taxa	90%CI
**DNA_C1vsC2**	**Nitrospira**	**27.29490368**
DNA_C1vsC2	Hyphomicrobium	6.965868301
DNA_C1vsC2	OM27_clade	12.252273
DNA_C1vsC2	Blastocatella	4.527497928
DNA_C1vsC2	Sphingomonas	7.832605989
DNA_C1vsC2	Methyloglobulus	9.972687698
DNA_C1vsC2	Woodsholea	11.51530212
DNA_C1vsC2	uncultured_Latescibacteria_bacterium	4.395130052
DNA_C1vsC2	Pseudomonas	20.78427096
DNA_C1vsC2	Variovorax	3.703893218
DNA_C1vsC2	Nitrosococcus	2.560913888
DNA_C1vsC2	Pedomicrobium	7.954590866
DNA_C1vsC2	uncultured	4.788737221
DNA_C1vsC2	ABS-19	6.385744045
DNA_C1vsC2	Nitrosomonas	2.349722921
DNA_C1vsC2	Rhizobacter	5.251468261
DNA_C1vsC2	CL500-29_marine_group	5.775774936
DNA_C1vsC2	Acidovorax	3.243152628
DNA_C4vsC3	Woodsholea	8.746445583
DNA_C4vsC3	uncultured_Latescibacteria_bacterium	6.922797297
DNA_C4vsC3	Blastocatella	5.900721972
DNA_C4vsC3	ABS_19	4.864921525
DNA_C4vsC3	Sphingomonas	8.427538926
DNA_C4vsC3	Azospira	3.467360657
DNA_C4vsC3	Pedomicrobium	11.24330224
DNA_C4vsC3	Hyphomicrobium	11.25563077
DNA_C4vsC3	Pseudomonas	21.77335039
DNA_C4vsC3	Nitrospira	8.999803853
DNA_C4vsC3	Nitrosomonas	5.042264724
DNA_C4vsC3	Nitrosococcus	2.410856048
DNA_C4vsC3	Methyloglobulus	7.6875397
DNA_C4vsC3	CL500-29_marine_group	10.03613588
DNA_C4vsC3	Acidovorax	6.612988123
DNA_C5vsC6	Woodsholea	34.61995489
DNA_C5vsC6	uncultured_Latescibacteria_bacterium	12.06266939
DNA_C5vsC6	uncultured	7.157708609
DNA_C5vsC6	Sphingomonas	11.87161387
DNA_C5vsC6	Pedomicrobium	27.32630925
DNA_C5vsC6	OM27_clade	35.22452854
DNA_C5vsC6	Nitrospira	69.06265794
DNA_C5vsC6	Nitrosococcus	6.604486979
DNA_C5vsC6	Methyloglobulus	21.34205684
DNA_C5vsC6	Hyphomicrobium	27.3900279
DNA_C5vsC6	CL500-29_marine_group	21.98392165
DNA_C5vsC6	Blastocatella	14.69858365
DNA_C8vsC7	Woodsholea	19.99953085
DNA_C8vsC7	OM27_clade	21.00440171

Table S1 – B Genus specific 90% CIs for the change in RNA buoyant density

Comparison	Taxa	90%CI
RNA.C1vsC2	Variovorax	0.010946202
RNA.C1vsC2	Methyloglobulus	0.012084529
RNA.C1vsC2	ABS-19	0.009321392
RNA.C1vsC2	CL500-29_marine_group	0.013380092
RNA.C1vsC2	Acidovorax	0.010681521
RNA.C1vsC2	OM27_clade	0.012539093
RNA.C1vsC2	Nitrosococcus	0.010281794
RNA.C1vsC2	Sphingomonas	0.011672409
RNA.C1vsC2	Rhizobacter	0.010244547
RNA.C1vsC2	Woodsholea	0.011844113
RNA.C1vsC2	Pseudomonas	0.010696879
RNA.C1vsC2	uncultured	0.010812986
RNA.C1vsC2	Nitrospira	0.013709626
RNA.C1vsC2	Blastocatella	0.012385466
RNA.C1vsC2	uncultured_Latescibacteria_bacterium	0.009498014
RNA.C1vsC2	Nitrosomonas	0.009555628
RNA.C1vsC2	Pedomicrobium	0.011050473
RNA.C1vsC2	Hyphomicrobium	0.010592564
RNA.C4vsC3	Woodsholea	0.008621579
RNA.C4vsC3	uncultured_Latescibacteria_bacterium	0.007414314
RNA.C4vsC3	Blastocatella	0.00661408
RNA.C4vsC3	ABS_19	0.008422849
RNA.C4vsC3	Sphingomonas	0.007734611
RNA.C4vsC3	Azospira	0.006711897
RNA.C4vsC3	Pedomicrobium	0.007508337
RNA.C4vsC3	Hyphomicrobium	0.00721053
RNA.C4vsC3	Pseudomonas	0.009573882
RNA.C4vsC3	Nitrospira	0.008457103
RNA.C4vsC3	Nitrosomonas	0.006827565
RNA.C4vsC3	Nitrosococcus	0.006299523
RNA.C4vsC3	Methyloglobulus	0.006913547
RNA.C4vsC3	CL500-29_marine_group	0.006162106
RNA.C4vsC3	Acidovorax	0.008380405
RNA.C5vsC6	Woodsholea	0.023065677
RNA.C5vsC6	uncultured_Latescibacteria_bacterium	0.022863257
RNA.C5vsC6	uncultured	0.021014205
RNA.C5vsC6	Sphingomonas	0.022086214
RNA.C5vsC6	Pedomicrobium	0.021504796
RNA.C5vsC6	OM27_clade	0.027101066
RNA.C5vsC6	Nitrospira	0.023046592
RNA.C5vsC6	Nitrosococcus	0.02328091
RNA.C5vsC6	Methyloglobulus	0.02270309
RNA.C5vsC6	Hyphomicrobium	0.020008472
RNA.C5vsC6	CL500-29_marine_group	0.022994518
RNA.C5vsC6	Blastocatella	0.021751132
RNA.C8vsC7	Woodsholea	0.000814697
RNA.C8vsC7	OM27_clade	0.001081158

Table S1-C Blast hits to the putative amoA sequences

Query	Description	Accession	e-value	score
**gene_274|GeneMark.hmm|128_aa|-|100|486**	hypothetical protein [Methyloferula stellata]	gi|519017702|ref|WP_020173577.1|	2.49E-49	169
**gene_238|GeneMark.hmm|188_aa|+|2|565**	hypothetical protein [Tistlia consotensis]	gi|1184553655|ref|WP_085121045.1|	2.01E-59	197
**gene_284|GeneMark.hmm|159_aa|-|152|631**	membrane protein [Cupriavidus sp. amp6]	gi|656005008|ref|WP_029046450.1|	1.20E-37	140
**gene_269|GeneMark.hmm|240_aa|-|1|720**	putative ammonia monooxygenase [Azospirillum brasilense]	gi|504008002|ref|WP_014241996.1|	4.71E-43	157
**gene_280|GeneMark.hmm|253_aa|+|1|759**	hypothetical protein [Hyphomicrobium sp. CS1BSMeth3]	gi|1119410285|ref|WP_072385832.1|	6.52E-72	232
**gene_235|GeneMark.hmm|197_aa|+|1|594**	ammonia monooxygenase [Phyllobacterium sp. YR531]	gi|495398156|ref|WP_008122856.1|	2.22E-66	215
**gene_248|GeneMark.hmm|175_aa|-|418|945**	hypothetical protein [Ramlibacter sp. Leaf400]	gi|946973621|ref|WP_055894610.1|	5.07E-52	177
**gene_271|GeneMark.hmm|262_aa|-|1|786**	hypothetical protein [Azospirillum brasilense]	gi|916533225|ref|WP_051140667.1|	4.79E-47	168
**gene_237|GeneMark.hmm|334_aa|-|3|1004**	hypothetical protein [Hyphomicrobium sp. CS1BSMeth3]	gi|1119410285|ref|WP_072385832.1|	4.89E-89	279
**gene_260|GeneMark.hmm|238_aa|+|2|718**	ammonia monooxygenase [Labrenzia alba]	gi|944196777|ref|WP_055678338.1|	6.67E-46	164
**gene_283|GeneMark.hmm|227_aa|+|399|1079**	hypothetical protein [Azospirillum brasilense]	gi|916533225|ref|WP_051140667.1|	2.28E-43	157
**gene_202|GeneMark.hmm|328_aa|+|575|1558**	hypothetical protein [Rhodoplanes sp. Z2-YC6860]	gi|1056597137|ref|WP_068028260.1|	4.65E-103	315
**gene_220|GeneMark.hmm|350_aa|-|179|1231**	ammonia monooxygenase [Polymorphum gilvum]	gi|503419284|ref|WP_013653945.1|	7.83E-69	227
**gene_205|GeneMark.hmm|291_aa|+|809|1681**	hypothetical protein [Caldimonas taiwanensis]	gi|1180938568|ref|WP_084362242.1|	6.46E-80	254
**gene_200|GeneMark.hmm|368_aa|-|567|1673**	hypothetical protein [Rhodoplanes sp. Z2-YC6860]	gi|1056597137|ref|WP_068028260.1|	6.38E-105	321
**gene_197|GeneMark.hmm|368_aa|+|44|1150**	hypothetical protein [Rhodoplanes sp. Z2-YC6860]	gi|1056597137|ref|WP_068028260.1|	6.38E-105	321
**gene_217|GeneMark.hmm|311_aa|-|636|1571**	membrane protein [Pseudogulbenkiania sp. MAI-1]	gi|635657271|ref|WP_024304138.1|	1.23E-75	243
**gene_223|GeneMark.hmm|380_aa|-|341|1483**	hypothetical protein [Hyphomicrobium sp. CS1BSMeth3]	gi|1119410285|ref|WP_072385832.1|	3.60E-127	378
**gene_227|GeneMark.hmm|366_aa|-|1878|2978**	hypothetical protein [Hyphomicrobium sp. CS1BSMeth3]	gi|1119410285|ref|WP_072385832.1|	7.66E-127	377
**gene_287|GeneMark.hmm|248_aa|-|2178|2924**	hypothetical protein [Rhizobiales bacterium CCH3-A5]	gi|1177645899|ref|WP_082736595.1|	2.94E-80	253
**gene_268|GeneMark.hmm|287_aa|-|2379|3242**	ammonia monooxygenase [Rhizobium sp. NT-26]	gi|918947167|ref|WP_052641815.1|	3.31E-36	140
**gene_209|GeneMark.hmm|352_aa|+|2469|3527**	membrane protein [Pseudogulbenkiania sp. MAI-1]	gi|635657271|ref|WP_024304138.1|	1.14E-83	265
**gene_257|GeneMark.hmm|292_aa|-|3418|4296**	ammonia monooxygenase [Rhizobium sp. NT-26]	gi|918947167|ref|WP_052641815.1|	9.85E-38	144
**gene_179|GeneMark.hmm|368_aa|-|4069|5175**	hypothetical protein [Rhodoplanes sp. Z2-YC6860]	gi|1056597137|ref|WP_068028260.1|	8.96E-108	328
**gene_277|GeneMark.hmm|271_aa|+|1|816**	AbrB family transcriptional regulator [Thauera sp. 27]	gi|489025714|ref|WP_002936124.1|	3.73E-39	148
**gene_233|GeneMark.hmm|350_aa|+|5216|6268**	ammonia monooxygenase [Polymorphum gilvum]	gi|503419284|ref|WP_013653945.1|	4.94E-67	223
**gene_246|GeneMark.hmm|318_aa|+|6379|7335**	ammonia monooxygenase [Azorhizobium caulinodans]	gi|501122003|ref|WP_012171155.1|	1.60E-48	174
**gene_196|GeneMark.hmm|336_aa|-|14681|15691**	membrane protein [Pseudogulbenkiania sp. MAI-1]	gi|635657271|ref|WP_024304138.1|	1.58E-85	270
**gene_124|GeneMark.hmm|359_aa|+|83125|84204**	hypothetical protein [Variovorax paradoxus]	gi|951108200|ref|WP_057597630.1|	1.15E-103	317
**gene_35|GeneMark.hmm|359_aa|-|30096|31175**	hypothetical protein [Variovorax paradoxus]	gi|951108200|ref|WP_057597630.1|	1.15E-103	317
